# Endothelial cells of pulmonary origin display unique sensitivity to the bacterial endotoxin lipopolysaccharide

**DOI:** 10.14814/phy2.15271

**Published:** 2022-04-19

**Authors:** Sofia K. H. Morsing, Eveline Zeeuw van der Laan, Anne‐Marieke D. van Stalborch, Jaap D. van Buul, Alexander P. J. Vlaar, Rick Kapur

**Affiliations:** ^1^ Molecular Cell Biology Lab Department Molecular Hematology Sanquin Research and Landsteiner Laboratory Amsterdam UMC University of Amsterdam Amsterdam The Netherlands; ^2^ Department of Experimental Immunohematology Sanquin Research and Landsteiner Laboratory Amsterdam UMC University of Amsterdam Amsterdam The Netherlands; ^3^ Leeuwenhoek Centre for Advanced Microscopy (LCAM) Section Molecular Cytology at Swammerdam Institute for Life Sciences (SILS) University of Amsterdam Amsterdam The Netherlands; ^4^ Department of Intensive Care Amsterdam UMC, location AMC Amsterdam The Netherlands

**Keywords:** ARDS, endothelial cells, LPS, TRALI

## Abstract

Acute respiratory distress syndrome (ARDS) is a major clinical problem without available therapies. Known risks for ARDS include severe sepsis, SARS‐CoV‐2, gram‐negative bacteria, trauma, pancreatitis, and blood transfusion. During ARDS, blood fluids and inflammatory cells enter the alveoli, preventing oxygen exchange from air into blood vessels. Reduced pulmonary endothelial barrier function, resulting in leakage of plasma from blood vessels, is one of the major determinants in ARDS. It is, however, unknown why systemic inflammation particularly targets the pulmonary endothelium, as endothelial cells (ECs) line all vessels in the vascular system of the body. In this study, we examined ECs of pulmonary, umbilical, renal, pancreatic, and cardiac origin for upregulation of adhesion molecules, ability to facilitate neutrophil (PMN) trans‐endothelial migration (TEM) and for endothelial barrier function, in response to the gram‐negative bacterial endotoxin LPS. Interestingly, we found that upon LPS stimulation, pulmonary ECs showed increased levels of adhesion molecules, facilitated more PMN‐TEM and significantly perturbed the endothelial barrier, compared to other types of ECs. These observations could partly be explained by a higher expression of the adhesion molecule ICAM‐1 on the pulmonary endothelial surface compared to other ECs. Moreover, we identified an increased expression of Cadherin‐13 in pulmonary ECs, for which we demonstrated that it aids PMN‐TEM in pulmonary ECs stimulated with LPS. We conclude that pulmonary ECs are uniquely sensitive to LPS, and intrinsically different, compared to ECs from other vascular beds. This may add to our understanding of the development of ARDS upon systemic inflammation.

## 1 INTRODUCTION

Acute respiratory distress syndrome (ARDS) is characterized by the onset of hypoxemic respiratory failure due to inflammation in the lungs and the development of pulmonary edema. In severe cases, ARDS may progress to multi‐organ failure, the leading cause of death in the ICU, and survivors often end up with permanent or long‐term morbidity (Bellani et al., 2016; Mayr et al., 2006). Causes of ARDS include bacterial infections, such as sepsis and pneumonia, viral infections such as SARS‐CoV‐1 and 2, or other causes such as trauma, pancreatitis or transfusion‐related acute lung injury (TRALI). In pneumonia and coronavirus infections, the lungs are the primary sites of infection, directly causing lung injury. Other conditions, however, such as sepsis, pancreatitis, and TRALI, are systemic and indirectly cause acute lung injury. It is unclear why particularly the lungs are so severely targeted compared to other organs upon systemic triggers. In experimental models of sepsis, intra‐peritoneal injections of lipopolysaccharide (LPS) are often used, resulting in lung injury (Aslan et al., 2017; Korneev, 2019). Experimental models of TRALI in specific pathogen free‐housed mice require similar priming with low‐dose LPS for significant induction of lung injury (Kapur et al., 2019; Kapur et al., 2018; Looney et al., 2009), based on the suggested involvement of the gut microbiota in driving TRALI responses (Kapur et al., 2018).

Endothelial cells (ECs) form the barrier between blood and tissue throughout the cardiovascular system, preventing plasma leakage and uncontrolled trans‐endothelial migration (TEM) of immune cells. Certain inflammatory stimuli, including LPS, activate ECs to upregulate surface expression of inflammatory adhesion molecules such as Intracellular‐Adhesion Molecule‐1 (ICAM‐1; CD54), Vascular Cell Adhesion Molecule‐1 (VCAM‐1; CD106), and Endothelial‐selectin (E‐selectin; CD62; ELAM‐1). These molecules are necessary for the capture and TEM of immune cells from the blood into tissue (Morsing et al., 2018). LPS activation of ECs additionally leads to the release of immune‐cell activating and recruiting cytokines such as IL‐1β, IL‐6, IL‐8, and TNF (Morsing et al., 2018). Under baseline conditions, ECs normally prevent plasma leakage also during TEM of immune cells (Heemskerk et al., 2016), but in ARDS pulmonary ECs are targeted, dysregulating their protective function. As a result, plasma and immune cells fill the alveoli, disrupting O_2_ gas exchange and damaging the lung tissue. In this study, we tested the hypothesis that pulmonary ECs may display increased sensitivity to LPS, compared to ECs of different organ origin, using an in vitro human primary EC model of systemically‐induced ARDS. We found that pulmonary ECs are uniquely sensitive to LPS, show an increased upregulation of critical adhesion molecules and barrier sensitivity to LPS. Using a proteomic screen, we found the junctional Cadherin‐13 to be highly expressed on lung ECs. Reducing Cadherin‐13 in lung ECs impaired LPS‐induced neutrophil TEM, suggesting that Cadherin‐13 may be a potential target to treat ARDS‐mediated lung‐specific pathologies.

## 2 MATERIALS AND METHODS

### 2.1 Antibodies

IF: mouse monoclonal against ICAM‐1 clone BBIG‐ I1(11C81) (R&D), chicken α‐mouse‐AF488 (Invitrogen), mouse monoclonal against VE‐cadherin (clone 55‐7H1, BD) conjugated with Alexa647. Western blot: CDH13 (R&D), CDH5 (clone TEA, Beckann Coulter), β‐actin‐HRP (clone AC15, Sigma Aldrich), rabbit polyclonal ICAM‐1 (Santa Cruz). Flow cytometry: BrilliantViolet‐510‐conjugated PECAM‐1 [WM59] (BD Biosciences), AlexaFluor‐647‐conjugated VE‐Cadherin [55‐7H1] (BD Biosciences), FITC‐conjugated ICAM‐1 [BBIG‐I1] (Biolegend), PE‐conjugated VCAM‐1 [51‐10C9] (BD Biosciences), E‐Selectin [68‐5H11] (BD Biosciences), AlexaFluor‐488‐ conjugated TLR4 clone HTA125 (Invitrogen).

### 2.2 Cell culture

Human microvascular endothelial cells (HMVECs) of pulmonary, renal, and pancreatic origin were purchased from PeloBiotech. Human umbilical vein endothelial cells (HUVEC), cardiac and additional pulmonary HMVEC were purchased from Lonza. All cells were cultured on fibronectin (FN)‐coated dishes in EGM‐2MV medium, supplemented with 5% FCS (PeloBiotech) and were age and gender matched as much as possible. Cells were cultured at 37°C and 5% CO_2_, used at passages 5–11 and controlled for endothelial markers after the end experiment. LPS from *Escherichia coli* [O55:B5] (Sigma‐Aldrich) diluted in PBS was added as indicated in the figures. Controls were treated with the same volume as LPS‐treated cells with PBS only. All cells were treated with 10 ng/mL of LPS for 5 h, unless otherwise indicated. Where indicated, experiments with short hairpins containing lenti‐Virus‐Like‐Particles (VLPs) of interest were performed at least 96 h after transduction.

### 2.3 Immunofluorescent staining

ECs subjected to flow assay were fixed in 3.7% (vol/vol) formaldehyde in PBS supplemented with 1 mM CaCl_2_, 0.5 mM MgCl_2_ (PBS^++^) for 20 min. PBS^++^ was added to preserve the junctions in experiments where the medium was absent. Concentrations of CaCl_2_ and MgCl_2_ equaled the concentration normally present in the medium. Next, cells were blocked with 2% BSA Fraction 5 (Serva) before incubation with primary and secondary antibodies. Between each incubation, cells were washed three times with PBS^++^. Finally, cells were kept in dH_2_O (Millipore) until imaging with a confocal laser‐scanning microscope (SP8; Leica; Aray; Zeiss).

### 2.4 Flow cytometry

Cells were detached with Accutase (GE‐Healthcare L11‐007) and harvested in PBS^++^ and 2% BSA Fraction 5 (Serva). For blocking, cells were placed on ice for 10 min. Cells were then pelleted and resuspended in FACS buffer (0.5% BSA: PBS^++^) at 2 × 10^6^ cells per milliliter. 50 µl of cell suspension (1 × 10^5^ cells) was added per well of a 96 wells plate. 50 µl of antibody solution in FACS buffer, at a concentration 2× the final concentration, was added to the wells containing cell suspension. The cells were incubated on ice, in the dark, for 30 min. Cells were pelleted and resuspended in FACS buffer, then measured on a LSR Fortessa (BD) cell analyzer using FACS Diva software. Cells were gated based on forward‐side scatter and PECAM‐1 and VE‐cadherin positivity, with a cell count of 1 × 10^4^ cells. FACS data was analyzed with FlowJo.

### 2.5 Electrical cell impedance sensing (ECIS) assay

Endothelial monolayer integrity was determined by measuring the electrical resistance using ECIS. Flow chamber electrode arrays (8W10E; Applied Biophysics, Troy, NY) were pretreated with 10 mM L‐cysteine (Sigma‐Aldrich) for 15 min at room temperature and subsequently washed twice with 0.9% NaCl. Wells were coated with fibronectin (Sanquin) in 0.9% NaCl for a minimum of 1 h at 37°C. Seeding density was 5 × 10^4^ cells/well. Continuous resistance measurements were performed at 37°C at 5% CO2 with the ECIS Zθ (Theta) system controller (Applied Biophysics). After the formation of a stable monolayer, cells were treated as indicated.

### 2.6 Generation of CDH13 KD short hairpins

Short hairpins directed against the coding DNA sequence of CDH13 were cloned to silence endogenous CDH13. In short, oligo’s containing the sense and antisense sequence of the short hairpin were dimerized and ligated in a pLKO.1‐puro backbone. The sequences for the short hairpins were identical to the validated sequences TRCN55544, TRCN55545, and TRCN55546 of the Sigma Predesigned shRNA library, with only alterations in the loop‐sequence. These constructs were used to produce VLPs, which were subsequently used to transduce pulmonary ECs. Control ECs were transduced with VLPs containing a short hairpin directed against non‐mammalian DNA. Production of VLPs day 1: 80% confluent HEK293T cells were transfected with constructs coding for the main structural viral proteins and for retrovirus‐specific enzymes (pHDM‐HgpM2 GAG/POL), coding for a post‐transcriptional regulator necessary for efficient GAG and POL expression (pRC‐CMV‐Rev1b REV), coding for the envelope of the Vesicular Stomatitis Virus (pHDMG‐G VSV ENV), coding for TAT 1B, which facilitates entry (pHDM‐TAT 1B), and a construct coding for a protein of interest. Trans*IT*‐LT1 (0.4 µl/cm^2^, Mirus Bio), was diluted in Optimem (10 µl/cm^2^, Life Technologies) and thoroughly mixed. To obtain a mastermix, viral constructs (44 ng/cm^2^ pHDMG‐G VSV ENV, 22 ng/cm^2^ pHDM‐HgpM2 GAG/POL, 22 ng/cm^2^ pRC‐CMV‐Rev1b REV, 22 ng/cm^2^ pHDM‐TAT 1B) were added to the diluted Trans*IT*‐LT‐1 transfection reagent. This master mix (10.4 µl/cm^2^) was added to a tube containing DNA coding for a protein of interest (289 ng/cm^2^). The transfection mix was left at room temperature for approximately 20 min before being added to the HEK293T cells. The cells were then placed back in the incubator at 37°C:5%CO_2_. Day 2: The medium containing the transfection mix was removed and replaced with approximately 100µl/cm^2^ of Dulbecco’s Modified Eagles Medium (DMEM, Life Technologies), supplemented with 2, 5–10% FBS, 200 mM L‐glutamine, 100 U/ml Penicillin/Streptomycin and 1mM Sodium Pyruvate. Cells were placed back in the incubator at 37°C:5%CO_2_. Day 3: DMEM containing the first 24 h of virus production was harvested and stored at 4°C. Approximately 100 µl/cm^2^ of fresh DMEM, supplemented with 2, 5–10% FBS, 200 mM L‐glutamine, 100 U/ml Penicillin/Streptomycin and 1mM Sodium Pyruvate, was added. Cells were placed back in the incubator at 37°C:5%CO_2_. Day 4: DMEM containing the second 24 h of virus production was harvested and pooled with the harvest of previous day. Cell debris was pelleted by centrifugation at 500 G for 5 min. The virus supernatant was filtered over a 0.45 µm pore filter and aliquots were prepared and stored at −80°C.

### 2.7 Neutrophil isolation

PMNs were isolated from whole‐blood derived from healthy donors. Whole blood was diluted (1:1) with 5% (v/v) TNC in PBS. Diluted whole blood was pipetted carefully on to 12.5 ml Percoll (room temperature) 1.076 g/ml. Tubes were centrifuged (Rotana 96R) at 800 G, slow start, low brake for 20 min. The bottom fraction containing PMNs was further processed by erythrocyte lysis in ice‐cold isotonic lysis buffer (155 mM NH_4_Cl, 10 mM KHCO_3_, 0.1 mM EDTA, pH 7.4 in Milli‐Q(Millipore). PMNs were centrifuged at 500 G for 5 min at 4°C and incubated again with lysis buffer for 5 min on ice. After another centrifugation at 500G for 5 min at 4°C, PMNs were washed once with PBS and centrifuged again at 500G for 5 min at 4°C before resuspension in HEPES medium (20 mM HEPES, 132 mM NaCl, 6 mM KCl, 1 mM CaCl_2_, 1 mM MgSO_4_, 1.2 mM K_2_HPO_4_, 5 mM glucose (all from Sigma‐Aldrich) and 0.4% (w/v) human serum albumin (Sanquin Reagents), pH7.4). PMNs count and purity were determined by a cell counter (Casy) and cells were kept at room temperature for no longer than 4 h before use.

### 2.8 Neutrophil transmigration under flow assay

ECs were plated at a concentration of 5 × 10^4^ cells per channel in fibronectin (FN)‐coated Ibidi μ‐slide VI0.4 (Ibidi) and cultured for 2–3 days. On the day of experiment, cells were treated with LPS or PBS. Freshly isolated neutrophils (PMNs), from healthy volunteers, were resuspended at 1 × 10^6^ cells/mL in HEPES medium pH 7.4. PMN was activated by incubating for 30 min at 37°C. Cultured ECs in Ibidi flow chambers were connected to a perfusion system and exposed to 0.5 mL/min HEPES medium pH 7.4, shear flow (0.8 dyn/cm^2^) for 5 min before injection of heat‐activated PMN into the perfusion system. Leukocyte‐endothelial interactions were recorded for 20 min at 0.2 frames/s by a Zeiss Observer Z1 or Zeiss Axiovert microscope. All live imaging was performed at 37°C in the presence of 5% CO_2_ at shear flow 0.8 dyn/cm^2^. Transmigrated PMNs were distinguished from those adhering to the apical surface of the endothelium by their transition from bright to phase‐dark morphology. A number of transmigrated PMNs was manually quantified using ImageJ and real‐time tracking was done with MTrackJ.

### 2.9 Statistical analysis

For statistical analysis between experimental groups, one‐way ANOVA, two‐way ANOVA or the student’s *t* test was used. A two‐sided *p* ≤ 0.05 was considered significant. Prism 8 (GraphPad Software, La Jolla, CA) was used for analysis.

## 3 RESULTS

### 3.1 Pulmonary HMVECs display increased ICAM‐1 expression and neutrophil‐TEM compared to HUVECs upon LPS stimulation

**FIGURE 1:**
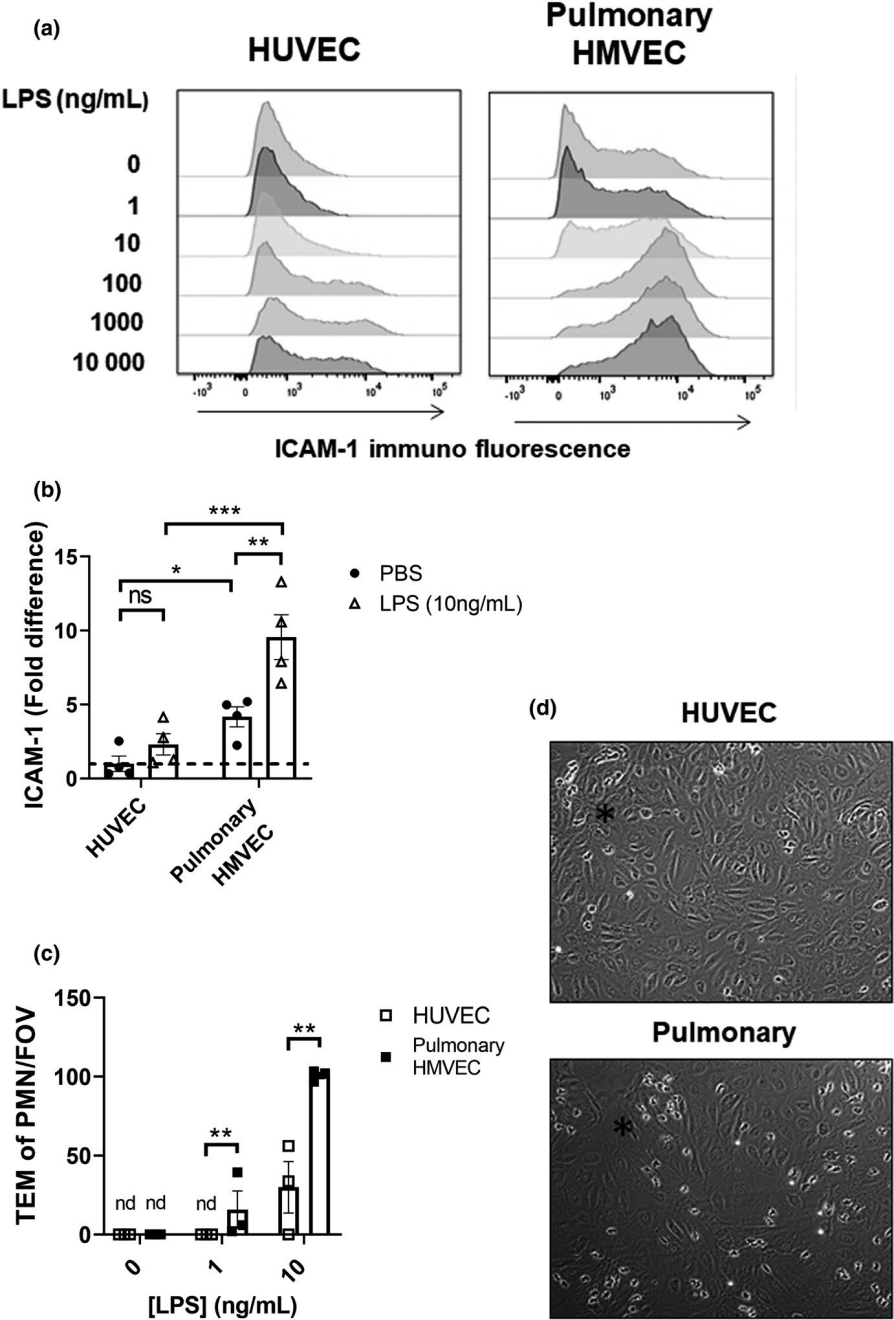
Pulmonary HMVECs display increased ICAM‐1 expression and a stronger and earlier response to LPS than HUVECs. (a) Histogram of ICAM‐1 distribution after 5 h LPS as measured with FACS. *Y*‐axis top to bottom represents increasing concentrations of LPS treatment of HUVEC and pulmonary HMVECs (pulmonary) as indicated. Condition 0 represents PBS CTRL treatment. *X*‐axis represents the increasing fluorescent intensity of ICAM‐1 measured with anti‐ICAM‐1‐FITC antibody. All cells were selected for PECAM‐1 and VE‐cadherin double positivity. (b) Quantification of ICAM‐1 surface expression after treatment of pulmonary HMVECs (pulmonary) and HUVEC with PBS (CTRL) or 10 ng/mL LPS as measured with FACS. All PBS‐treated HUVEC data points were divided by their average to indicate variation. All pulmonary HMVECs data points were divided by PBS‐treated HUVEC data points for fold difference. The dotted line indicates 1‐fold. ns = non‐significant, **p < 0.01, ***p < 0.001, *n* = 4. (c) Number of transmigrated neutrophils during flow TEM assay in HUVEC and HMVEC (Pulmonary) treated as indicated. Each data point represents average neutrophil TEM per field of view per condition. Three fields of view per channel were obtained during time‐lapse imaging, followed by tile scans with 25 additional fields of view per condition and replicate at the endpoint to determine the distribution of neutrophils. Bar represents an average of all experiments per condition combined. nd = not detected, **p < 0.01, error bars are presented as standard error of mean (SEM), *n* = 3. (d) Representative images from TEM assay. Black star indicates an area with a small cluster of transmigrated PMNs

In order to determine differences in LPS response between pulmonary Human Microvascular Endothelial Cells (HMVECs) and HUVECs, we first investigated adhesion molecule cell surface expression. Previously, we have used a high concentration of 1000 ng/mL LPS to elicit a maximum response in HUVECs (Morsing et al., 2020). To determine the sensitivity of pulmonary HMVEC and HUVECs to LPS, we performed a dose‐response experiment using FACS analysis measuring surface expression of ICAM‐1 (Figure 1a, Figure S1a) and VCAM‐1 (Figure S1a) at 5 h post‐treatment. ICAM‐1 and VCAM‐1 both followed the same pattern of upregulation, however, pulmonary HMVEC started upregulating the adhesion molecules already at an LPS concentration 10 times lower than HUVECs (Figure 1a,b, Figure S1a). Furthermore, the pulmonary HMVEC response to LPS plateaued at 100 ng/mL of LPS treatment, after which there was no additional increase in adhesion molecule upregulation with increased doses (Figure 1a, Figure S1a). Interestingly, pulmonary HMVECs expressed relatively high surface ICAM‐1 (4.169 ± 0.672) compared to HUVEC (1 ± 0.521) at baseline, that is, without any LPS stimulation. Following stimulation with 10 ng/mL of LPS, pulmonary HMVEC expressed about five times more (9.557 ± 1.511) surface ICAM‐1 than HUVEC (2.309 ± 0.720) (Figure 1b). Higher baseline ICAM‐1 expression in pulmonary HMVECs compared to HUVEC, and upregulation in response to LPS, was additionally confirmed with IF staining (Figure S1d,e) and in whole cell lysates with Western blotting (Figure S1b,c).

To determine to what extent pulmonary HMVECs and HUVECs would facilitate PMN trans‐endothelial migration (TEM) in response to LPS, we used a PMN‐TEM assay under physiological flow conditions in which the ECs form a confluent monolayer. Five to 6 h before the start of the TEM flow assay, ECs were stimulated with LPS or PBS (0 ng/mL LPS) as indicated. When treated with PBS, neither EC type facilitated TEM of PMNs (Figure 1c, with representative images from TEM assay shown in Figure 1d). However, already at 1 ng/mL of LPS treatment, pulmonary HMVECs started facilitating TEM with an average of 16 (±12) transmigration events per field of view (FOV). No transmigration events were observed in HUVECs at this concentration of LPS (Figure 1c). At 10 ng/mL of LPS, both cell types facilitated TEM, however, pulmonary HMVECs very efficiently (4‐fold increase) facilitated TEM compared to HUVECs (Figure 1c). When pulmonary HMVECs were treated with 100 ng/mL of LPS or higher, the EC monolayer was no longer intact and the flow assay was no longer feasible (data not shown).

### 3.2 Pulmonary HMVECs efficiently facilitate LPS‐induced neutrophil‐TEM compared to other types of HMVECs

**FIGURE 2:**
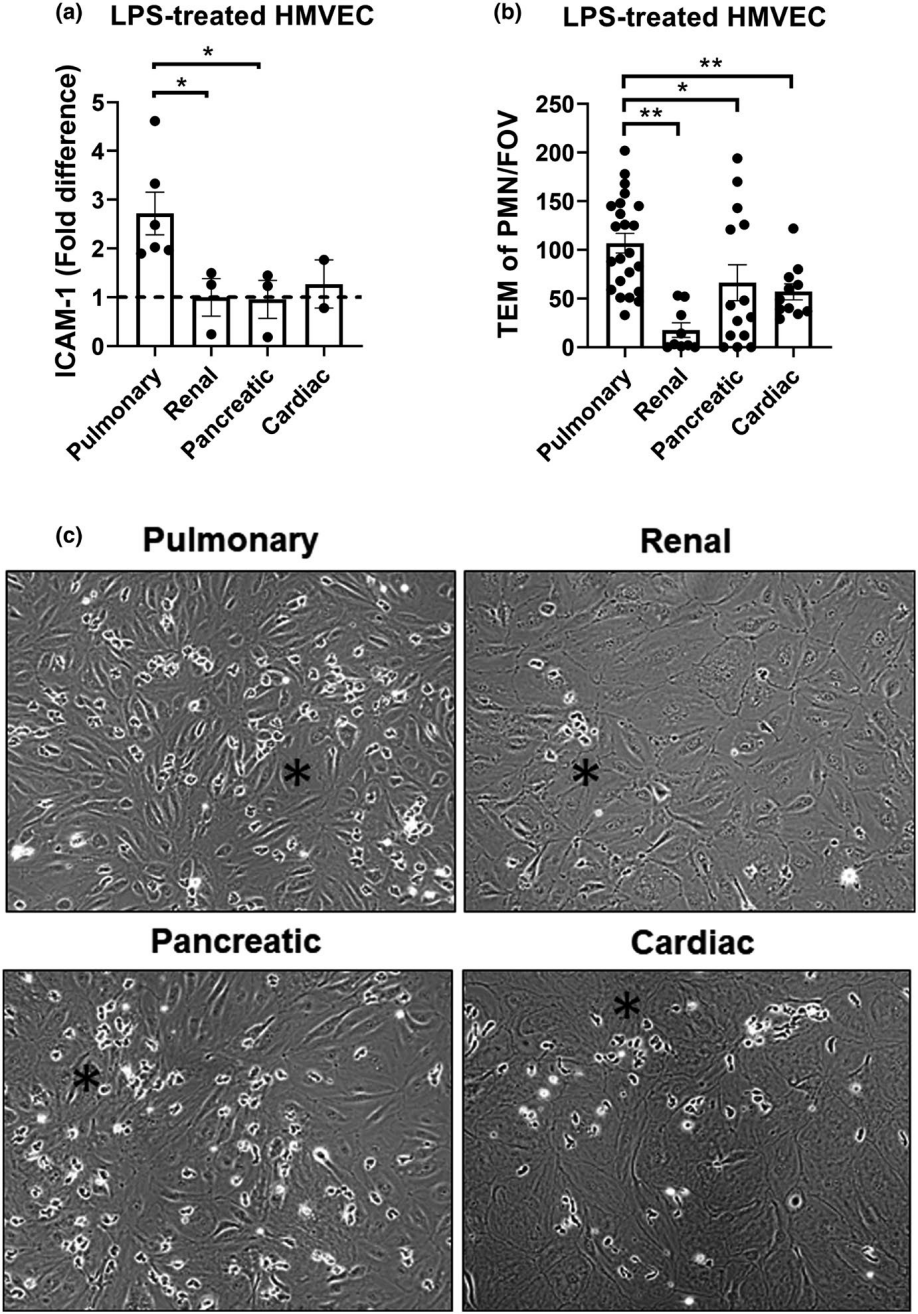
Pulmonary HMVECs show increased ICAM‐1 expression and efficiently facilitate LPS‐induced neutrophil TEM compared to other types of HMVECs. (a) Quantification of FACS data for ICAM‐1 at treatment with 10 ng/mL LPS. Renal HMVEC data points were divided by their own average to indicate variance. All other data points were divided by renal HMVEC data points to acquire fold difference. All cells had been selected for PECAM‐1 and VE‐cadherin double‐positivity. *p < 0.05, error bars are presented as standard error of mean (SEM), *n* = 3 except HCEC *n* = 2. (b) Number of transmigrated neutrophils during flow TEM assay in all organ microvascular ECs as indicated after 5 h treatment with 10 ng/mL LPS. Data points represent PMN‐TEM per field of view (FOV). Three fields of view per channel were obtained during time‐lapse imaging, followed by tile scans with 25 additional fields of view per condition and replicate at the endpoint to determine the distribution of neutrophils. Bars represent average TEM of PMN/FOV for all experiments. *p < 0.05, **p < 0.01, error bars are presented as standard error of mean (SEM), *n* = 3–6. (c) Representative images from TEM assay. Black star indicates an area with a small cluster of transmigrated PMNs

As HUVECs are ECs of fetal and venous origin, we next compared pulmonary HMVECs to more relevant ECs, that is, renal, pancreatic, and cardiac HMVECs. All EC types formed a confluent monolayer (Figure S2a). 10 ng/mL of LPS was used as the standard concentration for analysis of ICAM‐1 expression and TEM of PMN. Pulmonary ECs were the only EC type to have a significantly higher ICAM‐1 expression than the other microvasculars after 5 h LPS stimulation, which was of fold difference 2.72 (±0.44) from renal ECs (1 ± 0.39) (Pancreatic: 0.96 ± 0.39; Cardiac: 1.28 ± 0.50) (Figure 2a). Unfortunately, we were unable to increase n‐values for the cardiac HMVECs due to limited availability, thus significance could not be determined for these cells. Notably, cardiac HMVECs also expressed a high amount of adhesion molecules, albeit not as high as pulmonary HMVECs (Figure S2b). We also evaluated VCAM‐1 and E‐selectin expression between the different HMVECs in response to LPS treatment, and found that ICAM‐1 was a good indicator of adhesion molecule response, with both VCAM‐1 and E‐selectin being higher expressed in pulmonary HMVECs than other HMVEC types, to a similar fold‐increase of ICAM‐1 (Figure S2b).

We also determined the ability of the different HMVECs to facilitate PMN‐TEM after LPS stimulation, using the TEM flow assay. Pulmonary HMVECs facilitated significantly more PMN‐TEM (107 ± 10) compared to the other organ microvascular ECs (pancreatic: 66 ± 18; cardiac: 57 ± 8), with renal HMVECs facilitating the least amount (18 ± 7) (Figure 2b, with representative images from TEM assay shown in Figure 2c). Of note, experiments using cardiac cells in Figure 2b were finished prior to the studies in Figure 2a, which is why an *n* = 3 was able to be obtained in Figure 2b but not Figure 2a.

### 3.3 Resistance of pulmonary HMVEC cell‐cell junctions is LPS‐sensitive in contrast to other HMVEC types

**FIGURE 3:**
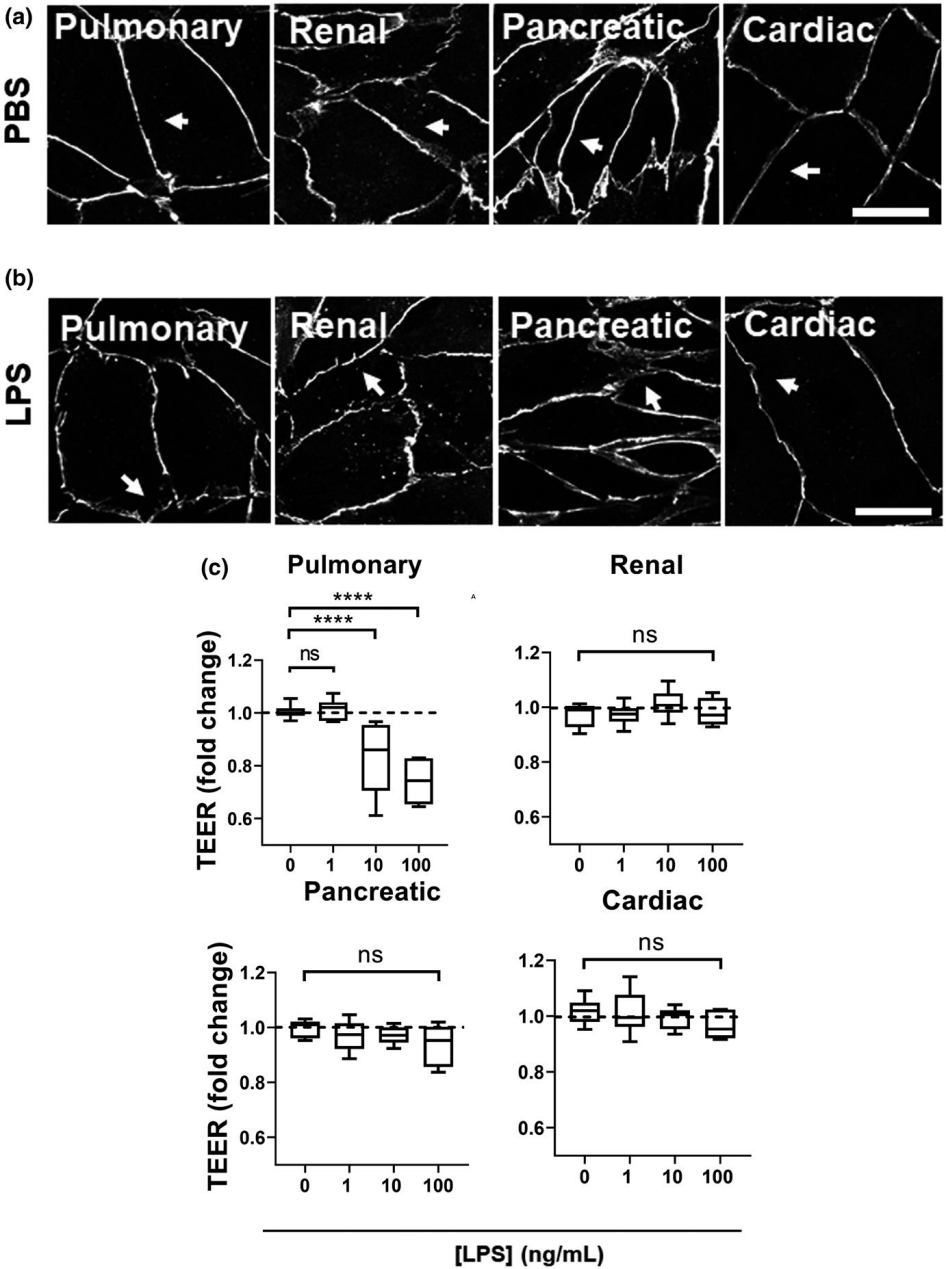
Resistance of endothelial cell‐cell junctions is dose‐dependently reduced by LPS in pulmonary HMVECs but not in other types of HMVECs. (a) Immunofluorescent images of ECs as indicated treated with PBS (CTRL) and stained for VE‐cadherin (white). Bar represents 20 µm. White arrows indicate linear junctions. (b) Immunofluorescent images of ECs as indicated treated with 10 ng/mL of LPS and stained for VE‐cadherin (white). Bar represents 20 µm. White arrows indicate jagged junctions between two cells. (c) Box‐plots of endothelial resistance indicating min to max values and average of all data points for all ECs as indicated. All data points were normalized to their own stabile resistance pre‐treatment with PBS or LPS. Dashed line indicates 1. TEER = Trans‐Endothelial Electrical Resistance. ****p < 0.0001, ns = non‐significant, *n* = 3–6

After establishing that pulmonary ECs most efficiently facilitated PMN‐TEM in response to LPS, we next analyzed the junction organization and EC barrier resistance, as a disrupted EC barrier leading to pulmonary edema is directly responsible for the development of acute lung injury in ARDS. To measure the resistance of the EC barrier, an Electrical Cell‐substrate Impedance Sensing (ECIS) assay was performed. Additionally, we analyzed the EC cell‐cell junction morphology of the different HMVECs after treatment with PBS (control) or LPS. Following PBS treatment, all HMVECs presented a generally linear organization of VE‐cadherin (Figure 3a). We and others have previously shown that linearity of junctions typically correlates with increased EC barrier resistance and vice versa (Ando et al., 2013; Morsing et al., 2020). The most linear junctions were observed in pulmonary and cardiac ECs (Figure 3a), which were additionally quantified (Figure S3a) as previously described (Morsing et al., 2020). Pulmonary and cardiac HMVECs indeed also presented the highest EC barrier resistance, as was measured with ECIS (Figure S3b). Upon LPS‐treatment, a slight junctional re‐organization was observed in all HMVECs (Figure 3b), which we have previously shown correlates with increased PMN‐TEM but not necessarily with junction resistance (Morsing et al., 2020). To determine if LPS treatment would affect the endothelial barrier, all HMVECs were grown to confluency and the monolayer barrier was measured using ECIS, followed by exposure to LPS in a titration. The box‐plots in Figure 3c represent 5–6 h of LPS stimulation to demonstrate the state of EC cell‐cell junctions at the time when PMN‐TEM experiments were performed, however, EC junction resistance was measured continuously over several days (data not shown). In contrast to the renal, pancreatic and cardiac HMVECs, pulmonary HMVEC junctions were dose‐dependently destabilized, that is, demonstrated a decrease in EC‐barrier resistance (Figure 3c, Figure S3c). Maximum resistance decrease was achieved between 5 and 8 h, after which junction integrity started to repair (Figure S3c).

These data suggests that pulmonary HMVECs are uniquely sensitive to barrier disruption due to LPS‐induced inflammation, while pancreatic, renal, and cardiac HMVECs maintain barrier integrity upon LPS challenge.

### 3.4 Pulmonary HMVECs do not display increased TLR4 or VE‐cadherin expression, but show increased CDH13 expression compared to other HMVEC types

**FIGURE 4:**
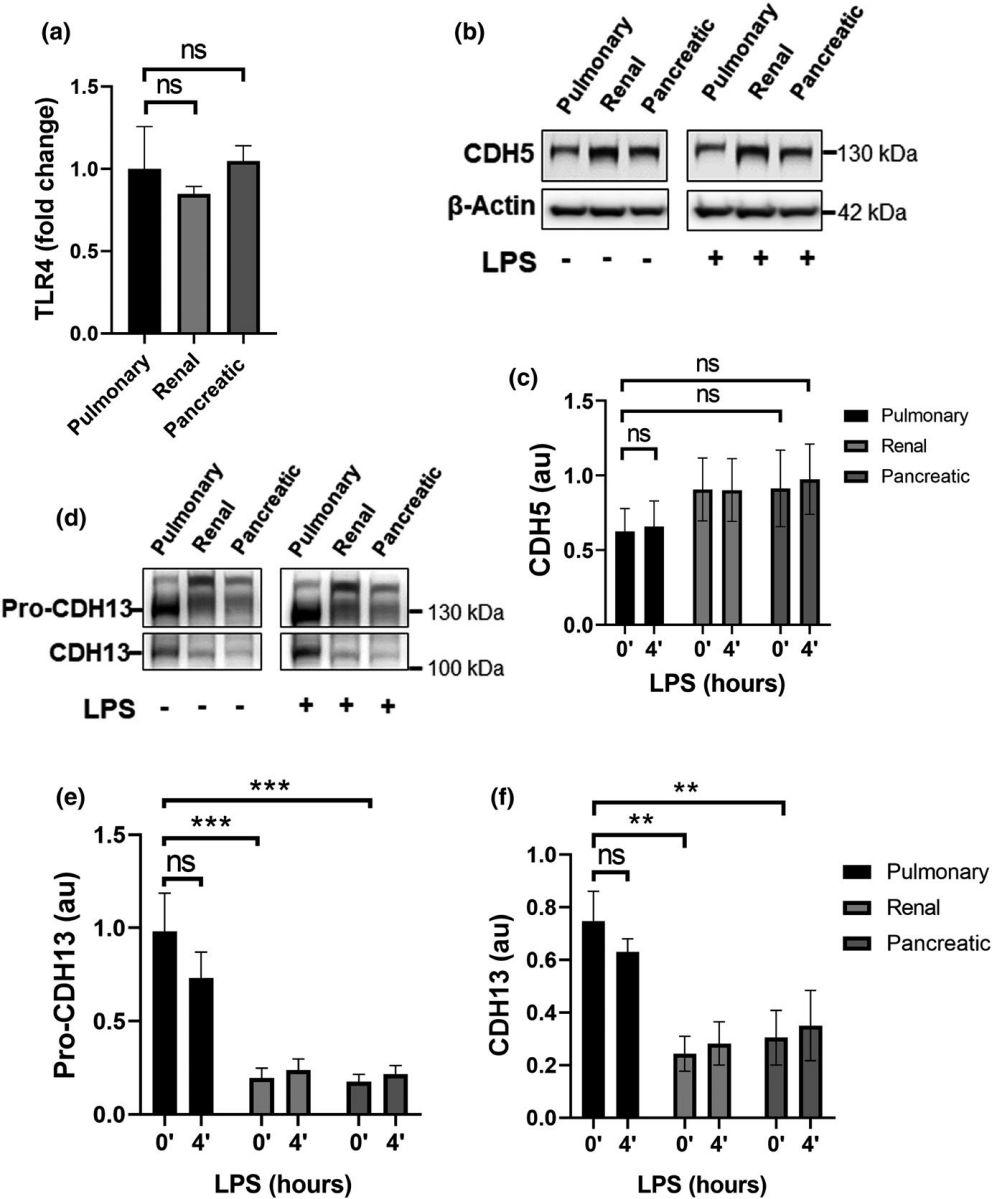
Pulmonary HMVECs do not display increased TLR4 surface expression compared to other types of HMVECs, but do display increased expression of CDH13. (a) Quantification of FACS data for TLR4 in untreated ECs as indicated. pulmonary HMVECs data points were divided by own average to indicate variance. All other data points were divided by pulmonary HMVECs data points to acquire fold the difference. All cells had been selected for PECAM‐1 and VE‐cadherin double‐positivity, ns, non‐significant, error bars are presented as the standard error of mean (SEM), *n* = 3. (b) Western blot of whole cell lysates from ECs as indicated treated with PBS (CTRL) or LPS and blotted for VE‐cadherin (CDH5) and β‐actin, as a loading control. (c) Quantification of Western blots presented in Figure 5(b). CDH5 units derived from ImageJ were divided by units for β‐actin. AU = arbitrary units, ns = non‐significant, error bars are presented as standard error of mean (SEM), *n* = 5. (d) Western blot analysis of whole cell lysates of ECs as indicated treated with PBS (CTRL) (0’) or LPS (4’) and incubated with antibodies against CDH13. CDH13 blot is the same as CDH5 in Figure 4b but developed on film with ECL. β‐actin blot is depicted in Figure 4(b). Images were analyzed with ImageJ. (e) Quantification of Western blots presented in Figure 4d. CDH13 units derived from ImageJ were divided by units for β‐actin. AU = arbitrary units, ns = non‐significant, **p < 0.01, ***p < 0.001, error bars are presented as standard error of mean (SEM), *n* = 5

We next investigated if the increased sensitivity of pulmonary HMVECs to LPS may be related to increased cell surface expression of toll‐like receptor (TLR)4. Surprisingly, using flow cytometry, TLR4 expression was found to be equal in all of the measured HMVECs; pulmonary, renal, and pancreatic HMVECs (Figure 4a). Additionally, we investigated the expression of VE‐cadherin (CDH5) using Western blotting, and found no significant differences between pulmonary, renal or pancreatic HMVECs, before or after LPS treatment (Figure 4b,c). These data were supported by a proteomics screen of pulmonary, renal and pancreatic HMVECs using mass‐spectrometry, in which no significant difference in expression of TLR4 or VE‐cadherin were found (data not shown). Unexpectedly, the proteomic analyses revealed a significant hit on cadherin‐13 (CDH13; H(eart)‐cadherin; T‐cadherin). CDH13 is not a very well‐known cadherin, but has been linked to GTPase signaling and regulation of the EC barrier (Andreeva et al., 2009; Philippova et al., 2005). CDH13 is also known as H‐cadherin, as it has been shown to be highly expressed in the heart (Jambusaria et al., 2020; Lee, 1996). Determining CDH13 expression of pulmonary, renal, and pancreatic ECs using Western blotting, we found baseline expression of pro‐CDH13 to be roughly three times higher in pulmonary HMVECs than that of renal or pancreatic HMVECs, and mature protein CDH13 on average twice the amount of renal or pancreatic ECs (Figure 4d). Upon LPS stimulation, there was a trend towards a decrease in pro‐CDH13 and CDH13 expression in pulmonary ECs following quantification of the Western blots (Figure 4e,f), however, this did not reach statistical significance and was not immediately visible upon blotting (Figure 4d).

### 3.5 CDH13 facilitates neutrophil‐TEM in pulmonary ECs upon LPS stimulation

**FIGURE 5:**
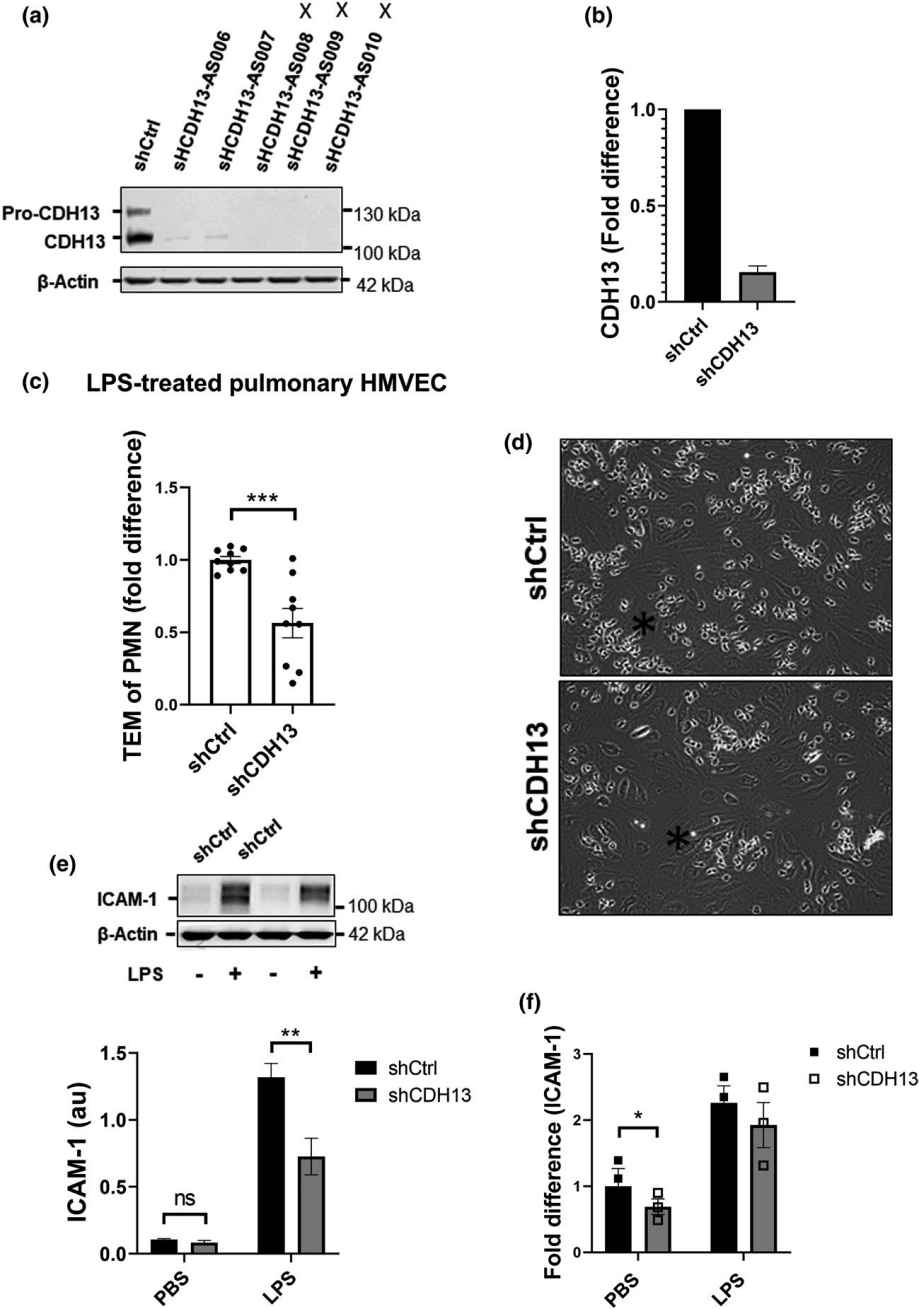
CDH13 aids neutrophil‐TEM in pulmonary HMVECs (a) Western blot analysis of whole cell lysates from pulmonary HMVECs after 96h from lentiviral transduction with short‐hairpin control (shCtrl) or short‐hairpin CDH13 (shCDH13) and incubated with antibodies against CDH13 and β‐actin. Images were developed on film using ECL. Images were analyzed with ImageJ. X marks the three constructs used for experiments. (b) Quantification of Western blot exampled in Figure 5a. shCtrl and shCDH13 units derived from ImageJ were divided by units for β‐actin to generate arbitrary units. All shCDH13 samples were divided by shCtrl which was set to 1 to indicate knock‐down efficiency. Error bar is presented as standard error of mean (SEM), *n* = 3 (c) Number of transmigrated neutrophils during flow TEM assay in pulmonary ECs after 5 h treatment with 10 ng/mL LPS. Data points represent PMN‐TEM per field of view (FOV). Three fields of view per channel were obtained during time‐lapse imaging, followed by tile scans with 25 additional fields of view per condition and replicate at the endpoint to determine the distribution of neutrophils. Bars represent average TEM of PMN/FOV for all experiments. ***p < 0.001, error bars are presented as standard error of mean (SEM), *n* = 3. (d) Representative images from TEM assay. Black star indicates an area with a small cluster of transmigrated PMNs. (e) Western blot analysis and quantification of whole cell lysates from pulmonary HMVECs after 96 h lentiviral transduction with short‐hairpin control (shCtrl) or short‐hairpin CDH13 (shCDH13), PBS or LPS stimulation for 20 h and incubated with antibodies against ICAM‐1 and β‐actin. Images were developed on film using ECL. Images were analyzed with ImageJ. ICAM‐1 units derived from ImageJ were divided by units for β‐actin to generate arbitrary units. AU = arbitrary units, ns = non‐significant, **p < 0.01, error bars are presented as standard error of mean (SEM), *n* = 3. (f) Flow cytometry data for ICAM‐1 in pulmonary ECs treated as indicated. PBS‐treated sh‐Ctrl pulmonary HMVECs data points were divided by their own average to indicate variance. All other data points were divided by PBS‐treated sh‐Ctrl pulmonary HMVECs data points to acquire fold difference. All cells had been selected for PECAM‐1 and VE‐cadherin double‐positivity, ns = non‐significant, error bars are presented as standard error of mean (SEM), *n* = 3

To find out if CDH13 may have a role in the increased sensitivity of pulmonary HMVECs for LPS, short hairpins directed against the coding DNA sequence of CDH13 were cloned to silence endogenous CDH13 in pulmonary HMVECs. These constructs were used to produce virus‐like particles, which were subsequently used to transduce pulmonary ECs. Control ECs were transduced with particles containing a short hairpin directed against non‐mammalian DNA. Western blot analysis confirmed a roughly 85% efficiency in CDH13 silencing in ECs compared to control levels at 96 h post‐infection (Figure 5a,b). Although all pre‐validated short‐hairpins achieved good CDH13 protein knockdown as determined by WB, their actual silencing efficacy may have varied as ECIS measurements showed consistent variance in effects on the resistance between the three shCDH13, with AS010 exhibiting the most profound effects (Figure S4a). All three short‐hairpins did indicate a prominent role for CDH13 in upholding junction resistance. To determine the effects of CDH13 KD on neutrophil TEM in LPS‐stimulated pulmonary HMVECs, shCtrl and shCDH13 treated pulmonary HMVECs were seeded in the flow channels, and a flow TEM assay was performed after 5–6 h of treatment with 10 ng/mL of LPS. Pulmonary ECs that were deficient for CDH13 showed an average of 50% reduction in TEM efficiency compared to control pulmonary ECs (Figure 5c, with representative images from TEM assay shown in Figure 5d), indicating that CDH13 plays an important role in facilitating PMN‐TEM in pulmonary ECs in response to LPS stimulation. Interestingly, the variance in PMN‐TEM of the shCDH13 treated ECs was once again short hairpin specific, with AS010 representing all the lowest data points and AS008 the highest.

Finally, we investigated ICAM‐1 expression in CDH13‐silenced pulmonary ECs versus controls. Western Blot analysis demonstrated a significant decrease of whole cell ICAM‐1 in LPS‐treated pulmonary ECs deficient in CDH13 (Figure 5e). Analysis of ICAM‐1 surface expression with flow cytometry indicated a decreased ICAM‐1 surface expression in PBS‐treated samples and a clear decreased trend towards LPS‐treated samples lacking CDH13 (Figure 5f).

## 4 DISCUSSION

ARDS is a major clinical problem, where the pulmonary endothelium becomes damaged by mechanisms that are incompletely understood. Why systemic inflammation triggers such a specific pulmonary response is currently unknown. In this study, we investigated the hypothesis that, compared to other types of endothelial cells, the pulmonary endothelium is more at risk for adverse events related to ARDS such as facilitation of PMN‐TEM and weakening of the endothelial barrier. We show that pulmonary ECs are uniquely sensitive to LPS compared to ECs of other organs. Our data may help to understand why patients develop ARDS secondary to inflammation outside the lungs. In lieu of inflammatory stimuli, pulmonary ECs displayed the highest constitutive expression of ICAM‐1 of all EC types measured. ICAM‐1 has been shown to be uniquely important for leukocyte trapping via LFA‐1 in the lung (Lehmann et al., 2003), thus this finding suggests that the pulmonary endothelium is already primed for risk of adverse cellular immune reactions targeting the endothelium. Indeed, particularly the aggregation of PMNs has been shown to determine the adverse effects in an in vivo model of sepsis‐induced ARDS (Park et al., 2019).

Also in TRALI inflammatory triggers like LPS prime the recipient, and blood transfusion subsequently triggers, through incompletely understood mechanisms, pulmonary PMN accumulation and PMN‐dependent EC damage (Semple et al., 2019). Together, this results in pulmonary edema and the development of TRALI (Semple et al., 2019). Interestingly, a recent study found that decreased endothelial NO production—a hallmark of dysfunctional endothelium—enhanced ICAM‐1 binding of leukocytes within 30 min, without altering protein expression (Gao et al., 2018). This may contribute to the predisposition of patients with underlying conditions, such as chronic alcohol abuse, obesity, and chronic kidney disease to develop respiratory failure during conditions such as TRALI or sepsis. In our study, upon stimulation with LPS at 1 ng/mL, an upregulation of adhesion molecules could already be seen in pulmonary HMVECs, and PMN‐TEM was demonstrated to be enhanced at this concentration. PMN‐TEM was not facilitated in HUVECs stimulated with 1 ng/mL of LPS, nor in renal or pancreatic HMVECs. Upon challenge with 10 ng/mL of LPS, all ECs responded by upregulating adhesion molecules and facilitating PMN‐TEM, however, pulmonary ECs facilitated up to four‐fold more PMN‐TEM.

Our findings match in vivo data of two recent studies where LPS was administered in experimental mouse models of hemorrhagic shock and sepsis, and mRNA expression of ECs from different vascular beds were investigated. In 2019, Jongman et al. measured mRNA of adhesion molecules ICAM‐1, VCAM‐1, and E‐selectin in pulmonary, cardiac, renal, and hepatic ECs, at baseline and following hemorrhagic shock or endotoxemia in WT versus Tie‐2 deficient mice (Jongman et al., 2019). As in our human primary cell model, their data also showed the highest expression of ICAM‐1, VCAM‐1, and E‐selectin in pulmonary ECs at baseline, which was unaltered by partial deletion of angiopoietin receptor Tie‐2, which has been linked to many endothelial functions, including upkeep of the endothelial barrier (Akwii et al., 2019; Aslan et al., 2014; Braun et al., 2020; Jongman et al., 2019). Following LPS stimulation, all ECs upregulated adhesion molecules, which were not attenuated in pulmonary ECs by partial deletion of Tie‐2. Jambusaria et al. performed an extensive gene expression profile for brain, cardiac and pulmonary ECs during homeostasis and after LPS administration at 6 h and 1 week. They found pulmonary ECs to have a specifically high expression profile of genes related to immune function such as leukocyte cell‐cell adhesion and leukocyte migration, T‐cell activation, and regulation of immune system processes. Furthermore, following LPS stimulation, pulmonary ECs exhibited the most profound dysregulation of core endothelial genes (Jambusaria et al., 2020). Our data using HMVECs complements their findings and highlights the functional consequences of this expression profile through analyses of PMN‐TEM and endothelial barrier integrity. Strikingly, only pulmonary HMVECs significantly disrupted the endothelial barrier at 10 ng/mL of LPS, which may facilitate the development of pulmonary edema in vivo. While using primary human EC cell cultures can be considered a strength of our study, on the other hand caution is advised regarding donor variability. We therefore carefully matched age and gender and excluded EC‐donors with a history of smoking, as this may already prime the ECs at baseline. Additionally, it is well known that ECs separated from their natural environment lose their specificity and become more similar to each other per passage. In this study, we did not use the ECs beyond passage 10, after which they lose much of their endothelial phenotype as well as their response to stimuli.

A strength of this study, on the other hand, is that the experimental findings were still observed even after several passages in vitro, suggesting an intrinsic and well conserved mechanism in pulmonary ECs specifically, which is not influenced by surrounding cells or the microenvironment. To further understand the increased sensitivity to LPS for pulmonary ECs, we examined TLR4 (a receptor for LPS) EC‐surface expression prior to LPS stimulation. To our surprise, no significant differences in TLR4 surface expression between the different types of ECs were found. Thus, a higher baseline expression of TLR4 in pulmonary ECs compared to other organ ECs did not explain why these cells were more sensitive to lower doses of LPS. In another study, Scott and colleagues observed the highest increase of phosphorylated ERK (p‐ERK) after LPS treatment in pulmonary ECs compared to the other cells measured, whereas phosphorylated p65 (p‐p65) was unaltered by LPS treatment (Scott et al., 2013). This may indicate that the LPS sensitivity of pulmonary HMVECs we observed, may be primarily mediated via the TRAF6/ERK pathway. This will be interesting to address in subsequent studies.

While we found VE‐Cadherin to have neither increased expression in pulmonary ECs, nor was VE‐Cadherin decreased in response to LPS, we did identify a previously unappreciated role for CDH13. CDH13 was first observed to be increased in pulmonary HMVECs in our proteomic screen, and we subsequently validated this by Western blot analyses. LPS treatment demonstrated a decreasing trend in pro‐CDH13 and CDH13 expression in pulmonary HMVECs, but unfortunately, the results were inconclusive and could not be determined with flow cytometry analysis, as the antibody was not suitable for this. To obtain further insights into the functional role of CDH13 in the LPS‐sensitivity of pulmonary HMVECs, we next used three different pre‐validated short‐hairpins to silence CDH13 protein in pulmonary HMVECs. Although all three short hairpins were pre‐validated and showed good CDH13 knockdown on Western blot, it seemed AS010 exhibited the most profound effects in pulmonary ECs. CDH13‐silenced pulmonary ECs showed reduced junctional TEER on ECIS, in keeping with previous observations by Andreeva et al. (Andreeva et al., 2009) and significantly decreased PMN‐TEM compared to shCTRL‐treated ECs. Although many targets of CDH13 have likely not been identified, it is known that CDH13 activates small Rho‐GTPases such as RhoA and Rac (Andreeva et al., 2009; Philippova et al., 2005). It is possible that CDH13 plays dual roles in homeostasis and inflammation, promoting barrier resistance via Rac1 under baseline conditions, whilst enhancing stress‐fiber formation via RhoA in LPS‐stimulated ECs. Interestingly, we found that silencing pulmonary HMVEC‐CDH13 significantly decreased whole cell ICAM‐1 and demonstrated a decreased ICAM‐1 surface expression in the PBS‐treated group and a decreased trend in the LPS‐treated group. A Genome‐Wide Association Study in the Taiwanese population showed an association between CDH13 risk alleles and increased soluble ICAM‐1 (Wu et al., 2017), supporting a mechanistic interaction between CDH13 and ICAM‐1 expression. CDH13 has also been shown to be elevated in early atherosclerosis (Philippova et al., 2011), and ICAM‐1 has been found to be crucial for the development of atherosclerosis (Lawson & Wolf, 2009; Wu et al., 2017). Future studies should focus on confirming the mechanistic link between CDH13 and ICAM‐1 and to subsequently investigate opportunities of therapeutically targeting CDH13.

To summarize, our findings suggest that the intrinsic nature of pulmonary ECs, with a role for CDH13, may enable the increased sensitivity to LPS reflected by increased expression of adhesion molecules, induction of PMN‐TEM and enhanced disruption of the endothelial barrier, thereby contributing to the development of ARDS. In addition, our study underscores the importance of carefully considering which type of ECs are used for any in vitro study focusing on the mechanisms behind endothelial organ disease. This study provides a first step in understanding why lungs may be primarily targeted in ARDS due to systemic inflammatory triggers.

## ETHICS STATEMENT

All blood donor volunteers signed informed consent, under the rules and legislation in place within the Netherlands and maintained by the Sanquin Medical Ethical Committee. The rules and legislations are based on the Declaration of Helsinki (informed consent for participation of human subjects in medical and scientific research) and guidelines for Good Clinical Practice.

## CONFLICTS OF INTEREST

The authors declare no conflict of interest.

## AUTHOR CONTRIBUTIONS

Sofia K. H. Morsing, Eveline Zeeuw van der Laan, Anne‐Marieke D. van Stalborch performed the experiments; and Sofia K. H. Morsing, Jaap D. van Buul, Alexander P. J. Vlaar, and Rick Kapur designed and analyzed the experiments, wrote, revised, and approved the manuscript.

## Supporting information



Fig S1‐S4

Supplementary Material
