# Supplementary material for: Endothelial cells of pulmonary origin display unique sensitivity to the bacterial endotoxin lipopolysaccharide
Source: Physiol Rep. 2022 Apr 19;10(8):e15271. doi: 10.14814/phy2.15271 (PMC9017980; doi:10.14814/phy2.15271)

Supplemental Figure 1.

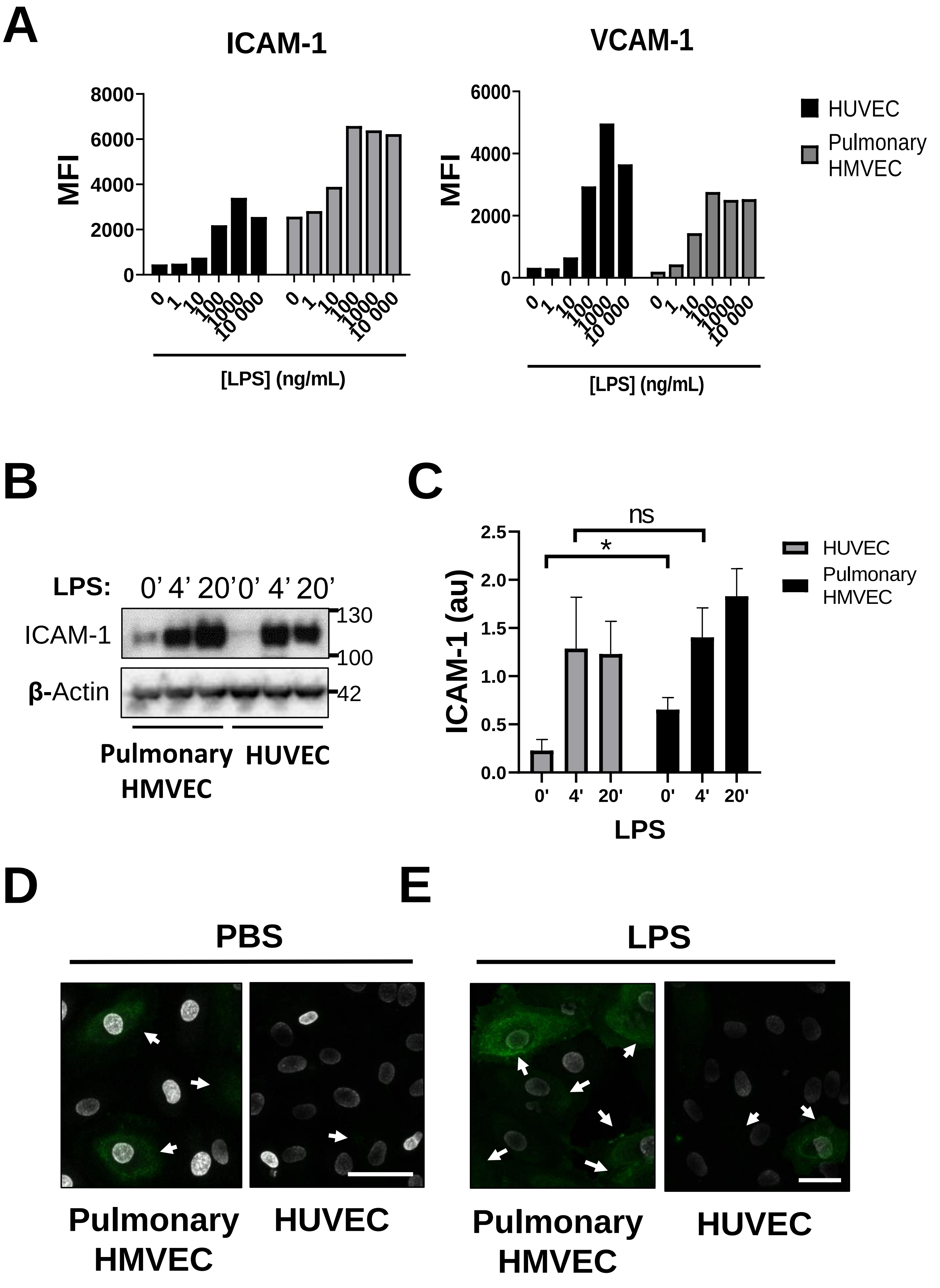

Supplemental Figure 2.

A

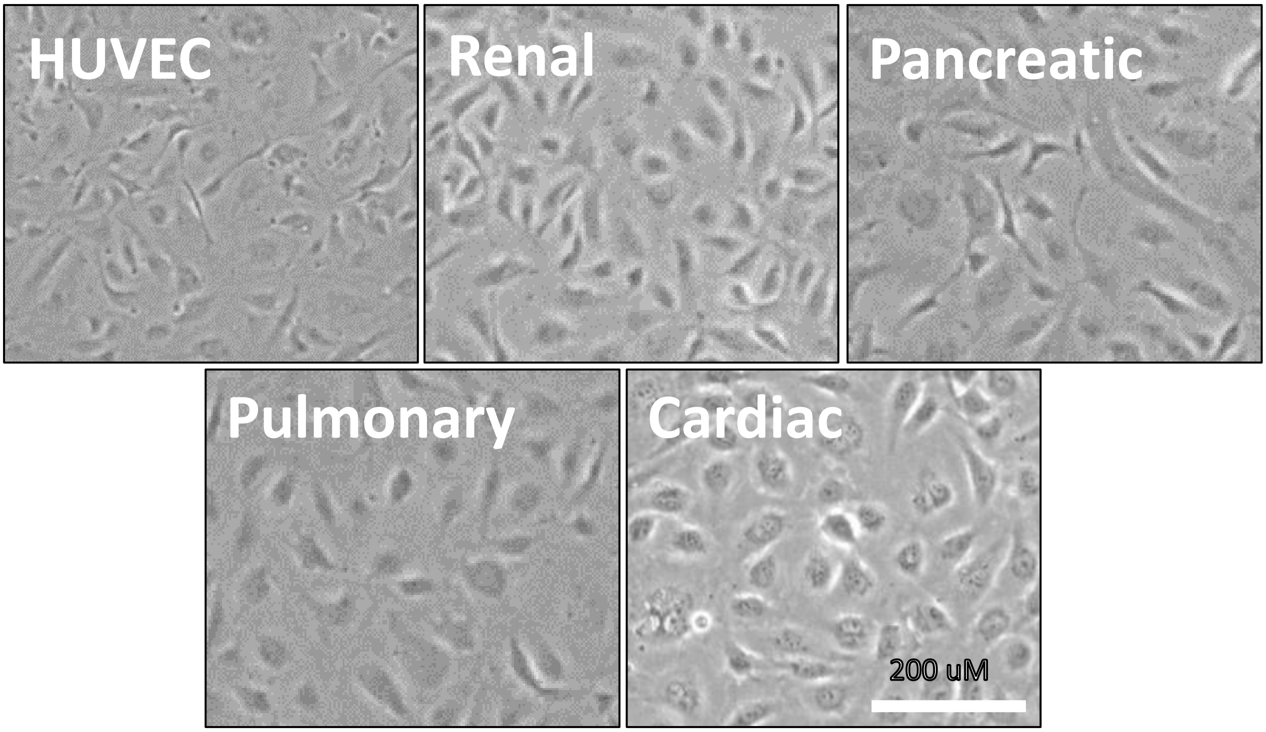

B

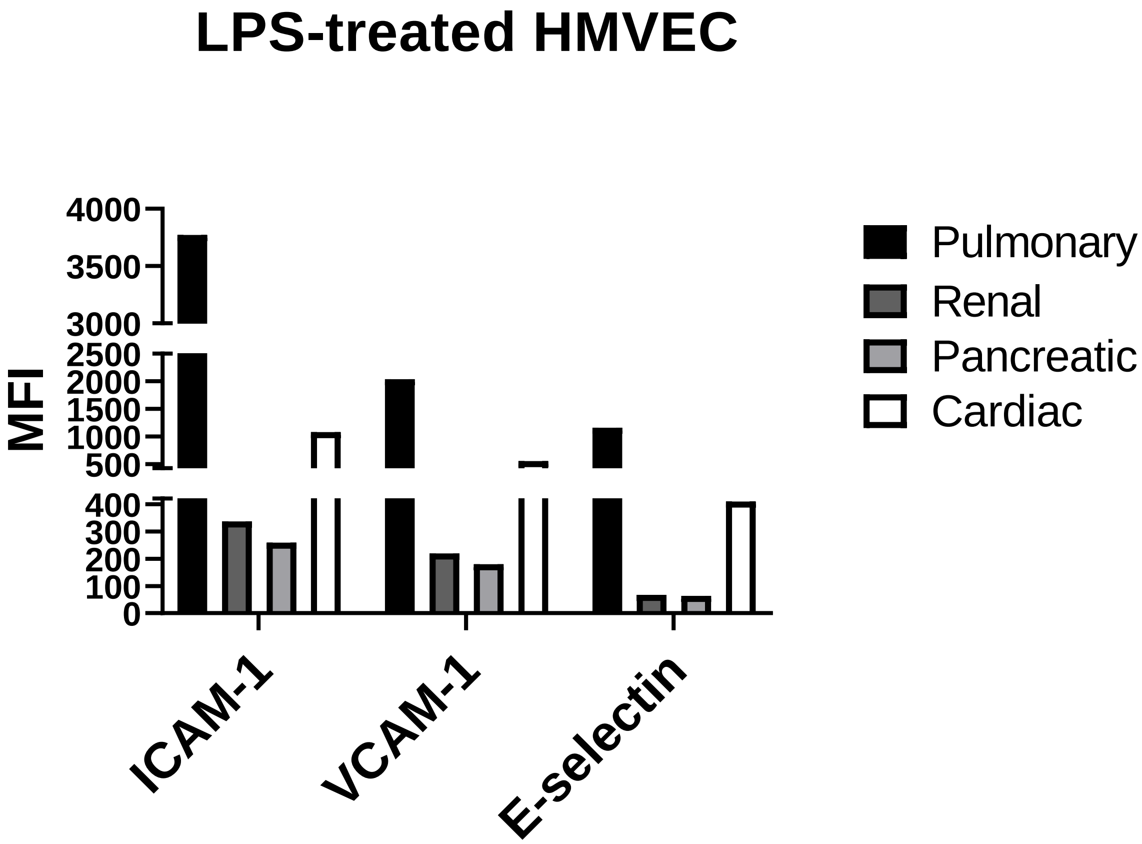

Supplemental Figure 3.

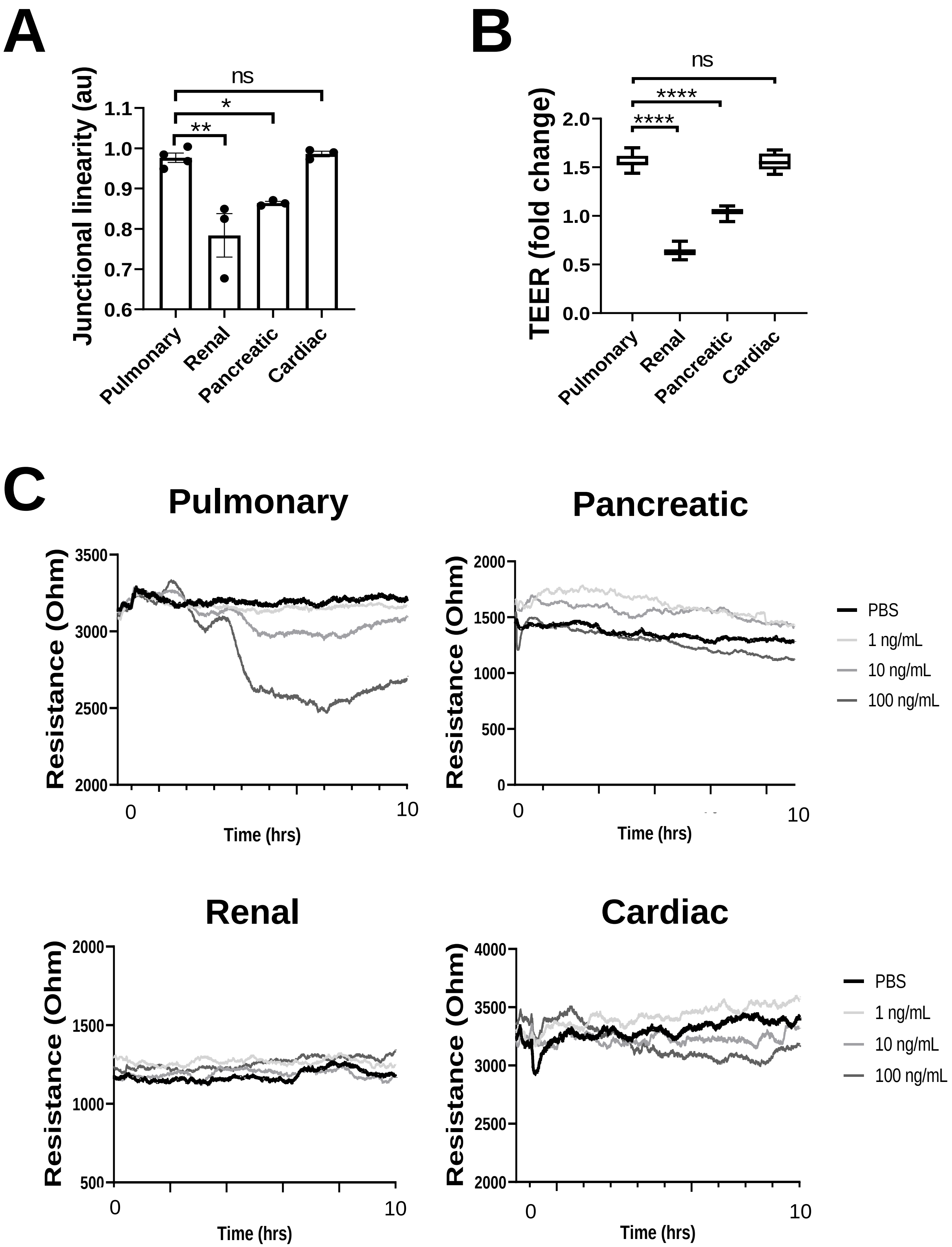

Supplemental Figure 4.

A

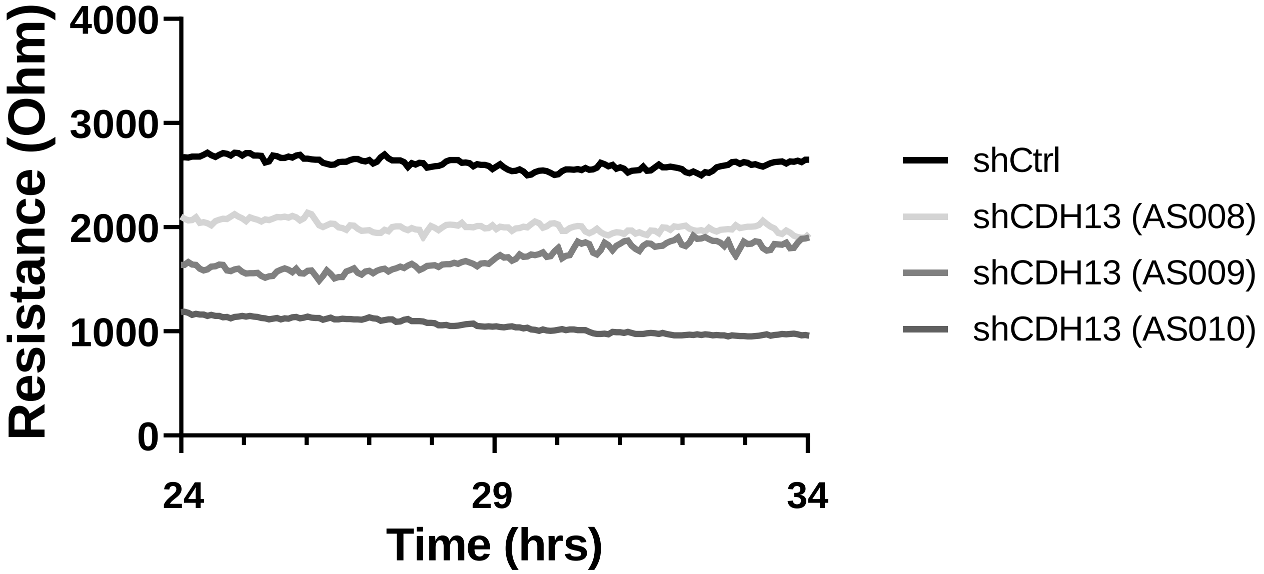

Supplement: Supplementary file 1 — Fig S1‐S4 [file PHY2-10-e15271-s002.pdf]
