# Supplementary material for: Endothelial cells of pulmonary origin display unique sensitivity to the bacterial endotoxin lipopolysaccharide
Source: Physiol Rep. 2022 Apr 19;10(8):e15271. doi: 10.14814/phy2.15271 (PMC9017980; doi:10.14814/phy2.15271)
Supplement: Supplementary file 2 — Supplementary Material [file PHY2-10-e15271-s001.docx]

**Figure legends**

**Figure S1.** **A)** MFI of ICAM-1 and VCAM-1 surface expression after a titration experiment treating HUVEC and Pulmonary HMVEC with PBS (0 ng/mL) or increasing amounts of LPS. **B)** Western blot analysis of ICAM-1 and β-actin expression in whole cell lysates of HPEC and HUVEC as indicated, PBS-treated (0’) or treated for 4 or 20h (4’ and 20’ respectively) with LPS prior to lysis. ICAM-1 blot is cropped at 130 and 95 kDa, β-actin blot is cropped at 55 and 36 kDa, as determined by a pre-stained protein standard. Bands were developed on film with ECL blotting reagent. **C)** Quantification of Western blots exampled in figure 1C. Intensity of bands was determined with ImageJ and units for ICAM-1 were divided by units for β-actin. AU = arbitrary units. Columns display SEM error bars, ns = not significant, * = p<0.05, n = 3. **D)** IF staining of ICAM-1 (green) of PBS-treated pulmonary HMVEC and HUVEC. White arrows indicate cells expressing ICAM-1. **E)** IF staining of ICAM-1 (green) of LPS-treated pulmonary HMVEC and HUVEC. White arrows indicate cells expressing ICAM-1.

**Figure S2. A)** EVOS phase/contrast images of HMVEC as indicated, white bar represents 200 µM **B)** MFIs of ICAM-1, VCAM-1 and E-selectin as indicated in an experiment where organ HMVECs were treated with 10 ng/mL of LPS.

**Figure S3. A)** Junctional linearity measurement of organ HMVECs treated with PBS and stained for VE-cadherin. At least 3 individual measurements were used per cell type. ns = not significant, * = p<0.05, ** = p<0.01 **B)** Boxplots of endothelial resistance indicating min to max values and average of all data points for organ HMVEC as indicated. All data points were normalized to the stable resistance of the pancreatic HMVEC. TEER = Trans-Endothelial Electrical Resistance. ****p<0.0001, ns=non-significant, n=3-6**. C)** Examples of continuous ECIS measurements from ECs treated with LPS as indicated at timepoint 0.

**Figure S4. A)** Example of continuous ECIS measurement from pulmonary ECs transduced with control or shCDH13 lentiviral particles. Timepoint 24h represents after 24h in ECIS machine, transduction of short hairpins was for minimum 96h.
